# Responsibility for Crashes of Autonomous Vehicles: An Ethical Analysis

**DOI:** 10.1007/s11948-014-9565-5

**Published:** 2014-06-11

**Authors:** Alexander Hevelke, Julian Nida-Rümelin

**Affiliations:** Lehrstuhl für Philosophie IV, Ludwig-Maximilians-Universität München, Geschwister-Scholl-Platz 1, 80539 Munich, Germany

**Keywords:** Ethics, Ethical, Moral, Autonomous car, Self-driving car, Responsibility, Liability, Duty to intervene

## Abstract

A number of companies including Google and BMW are currently working on the development of autonomous cars. But if fully autonomous cars are going to drive on our roads, it must be decided who is to be held responsible in case of accidents. This involves not only legal questions, but also moral ones. The first question discussed is whether we *should* try to design the tort liability for car manufacturers in a way that will help along the development and improvement of autonomous vehicles. In particular, Patrick Lin’s concern that any security gain derived from the introduction of autonomous cars would constitute a trade-off in human lives will be addressed. The second question is whether it would be morally permissible to impose liability on the user based on a duty to pay attention to the road and traffic and to intervene when necessary to avoid accidents. Doubts about the moral legitimacy of such a scheme are based on the notion that it is a form of defamation if a person is held to blame for causing the death of another by his inattention if he never had a real chance to intervene. Therefore, the legitimacy of such an approach would depend on the user having an actual chance to do so. The last option discussed in this paper is a system in which a person using an autonomous vehicle has no duty (and possibly no way) of interfering, but is still held (financially, not criminally) responsible for possible accidents. Two ways of doing so are discussed, but only one is judged morally feasible.

## Introduction

“*Cars crash. So too will autonomous vehicles, a new generation of vehicles under development that are capable of operating on roadways without direct human control.*” (Marchant and Lindor 2012) We can probably expect this assumption to be correct.^1^ This leads to a central legal question surrounding the use of fully autonomous cars: who should be made responsible if such a crash occurs? The present article will discuss this question from an ethical standpoint.

We will assume that it will be possible to design autonomous vehicles which cause fewer and less severe accidents than cars steered by the average driver. *“If autonomous vehicles have statistically more, or more severe, accidents than standard cars, then such vehicles will not be legally viable for widespread use.”* (Marchant and Lindor 2012) The same is probably true from a moral, political or economic perspective. Therefore, if autonomous cars prove less safe than human-driven ones, that would render the questions raised in this article moot.

## Responsibility of the Manufacturer

Holding the manufacturers responsible for any crash caused by the vehicle would probably be the most obvious solution. They are, after all *“ultimately responsible for the final product”* (Marchant and Lindor 2012): the vehicle including the system guiding it. If there is some flaw (or some design decision)^2^ in the system, which tends to cause accidents in certain situations, they probably knew or should have known about it but sold the defective autonomous cars anyway. Why should they not have to take responsibility?

The clearest answer is a practical one: if in the case of crashes involving autonomous vehicles the main responsibility were to be that of the manufacturers, *“the liability burden on the manufacturer may be prohibitive of further development.”* (Marchant and Lindor 2012) Of course, full legislative protection from liability would probably also have undesirable effects: “*it diminishes, if not eliminates, the incentives for manufacturers to make marginal improvements in the safety of their products in order to prevent liability.”* (Marchant and Lindor 2012) Could a partial liability be designed in such a way that the continuous development and improvement of autonomous vehicles would not be impeded but promoted? It seems likely, but this question would have to be discussed and answered elsewhere. An ethical analysis would not solve it.

There is, on the other hand, the question of whether we should try to promote the development of autonomous cars to begin with. In other words: *should* we try to design the liability for autonomous vehicles in such a way that it promotes their continuous development and improvement? Should such vehicles be allowed on our streets? These questions can be addressed through normative ethics. If there are good *moral* reasons for finding the development and introduction of autonomous cars to be desirable, this can produce a moral obligation for the state to fashion the legal responsibility for crashes of autonomous cars in a way which helps the development and improvement of autonomous cars.

There are many arguments which can be made in favour of or against the introduction of autonomous cars. Possible problems include privacy issues (Glancy 2012) and environmental harm from fully-autonomous vehicles, as these could lead to more vehicle-miles travelled (Elkind 2012). On the positive side, the introduction of autonomous cars might among other things enable the physically impaired, disabled or elderly to drive their own vehicles (Howard 2013). However, a thorough discussion of these additional issues would exceed the scope of this article.

We will therefore focus on one possible reason in favour of autonomous cars which (at least potentially) could be of tremendous moral weight: The development and widespread use of autonomous cars could cause a reduction of accidents, it could therefore save lives.^3^ Even if we are talking of a relatively small improvement like a reduction of 5 % it would save hundreds of lives a year in countries like the US in which deaths in road accidents go into the tens-of-thousands. This by itself seems like a powerful prima-facie reason to promote the development of autonomous cars (we would and should probably not be willing to sacrifice hundreds of people to improve the privacy of car-users or to avoid an increase of miles driven per year). But is it? There might be reasons to have doubts about the moral status of these saved lives:

“Let’s say that autonomous cars slash overall traffic-fatality rates by half. So instead of 32,000 drivers, passengers, and pedestrians killed every year, robotic vehicles save 16,000 lives per year and prevent many more injuries. But here’s the thing. Those 16,000 lives are unlikely to all be the same ones lost in an alternate world without robot cars. When we say autonomous cars can slash fatality rates by half, we really mean that they can save a net total of 16,000 lives a year: for example, saving 20,000 people but still being implicated in 4,000 new deaths. There’s something troubling about that, as is usually the case when there’s a sacrifice or “trading” of lives. The identities of many (future) fatality victims would change with the introduction of autonomous cars. Some victims could still die either way, depending on the scenario and how well robotic cars actually outperform human drivers. But changing the circumstances and timing of traffic conditions will likely affect which accidents occur and therefore who is hurt or killed. […] some current non-victims—people who already exist—would become future victims, and this is clearly bad.” (Lin 2013).

This objection, if it proved true, could be very powerful: The central function of a democratic, liberal state is to safeguard individual rights and liberties. Its central norms should not be consequentialist but deontological in nature and the interpretation of these norms is categorical, not hypothetical. A violation of some person’s fundamental rights cannot be legitimized on the basis of benefits for others, no matter how large. The normative order of a democracy should recognize individual rights, and the right to live protects every single individual against state decisions which might threaten them. To protect these fundamental individual rights is a goal of the state which should override any other. Other goals, including the interests of the majority, cannot be weighed against them. Of course, additional normative factors such as well-being or equality also play an important role. Nevertheless, the deontological character of a liberal democracy’s normative order manifests itself partly in preventing trade-offs when it comes to certain individual rights and liberties. The minimisation of collective risks therefore often comes into conflict with this constitutive element of any humane order and specifically of liberal democracies.^4^

However, is it plausible to make out a conflict of that kind in our case? Would the introduction of autonomous vehicles really sacrifice the interests of one group to safeguard the interests of the majority? The introduction of autonomous vehicles is quite different from the paradigm of trolley-cases.^5^ In contrast to the standard trolley-case, we should not focus on the actual damage done in the end, when we try to determine if a decision in favour of autonomous vehicles is in the interest of one of the affected parties. Instead, the risks at the time of the decision should become central. Whether or not the introduction of a new safety feature is in the interest of a person does not depend on whether or not the person in question does have an accident in the end or how bad it may come to be. It depends on whether the feature improves his chances of avoiding the accident or reduce possible damage. If an action is in the interest of a person, this is true irrespectively of what its consequences are. Let’s say he had to travel from Egypt to South Africa and it were rational to expect one form of traveling (e.g., using airplanes) to be significantly safer than another (e.g., going by boat). Assuming he were correspondingly advised by his friend to use the plane; this action remains prudent even if it turns out that the airplane crashes and his colleague, who took a boat, had a perfectly safe and pleasant journey. It remains prudent under the condition that his expectation was rational or that it was rational to follow the advice of the more experienced or better-informed friend. Even a very low probability that some event might occur is compatible with the fact that this event did in fact take place. The prudential choice has maximal expected value regarding the interest of the person in question. A decision is prudential if and only if its expected value with regard to a person’s personal interests is maximal at the time the decision is made, whatever its eventual consequences. Therefore, we judge an action to be in the interest of a person given the probabilities and given the preferential situation at the moment the decision takes place. It is a fallacy to take the real consequences of a decision into account when confronted with probabilistic phenomena. What counts when we decide if a possible action is in the interest of a person is the probability of that decision’s consequence, not the actual consequence itself.

The non-identity problem that Lin refers to in the above quotation is therefore only applicable to autonomous cars if there is an identifiable group of individuals whose *risks* might be increased by the introduction of autonomous cars. If that proves to be the case, it would certainly pose a problem. Otherwise, Lin’s concerns are unfounded.^6^ The introduction of autonomous cars would be no different (in this regard) to the introduction of already well-established safety features such as seatbelts or glued-in laminated windshields. There might be cases in which any of these safety features do more harm than good. A modern, glued-in windshield might, for example, delay rescue for a few critical moments, causing an accident victim to bleed to death. However, these tragic cases do not change the fact that having these features significantly improves the overall safety of those who use cars. Having them as part of the car is therefore in the interest of the users. Moreover, this is also true for those unlucky few who end up hurt because of them. The objection that the introduction of autonomous vehicles would sacrifice a smaller group for the good of a larger one is therefore unfounded.

The consequentialist tries to reduce the diversity of morally relevant factors including duties, rights, principles etc. to one single principle: one should maximize the good. There are many reasons to reject such a reductionist scheme. (Nida-Rümelin 1995) However, a teleological rationale can still provide powerful moral reasons, especially when the good to be promoted is as important as in this case. Death and injury caused by accidents *are* an evil, and the protection of its citizens from such harm *is* a central task of the state. If the introduction of autonomous vehicles might reduce the yearly toll in death and injury exacted by road traffic even by a small degree, that would constitute a powerful moral reason in favour of promoting their development—which includes trying to design car manufacturers tort liability in such a way that the development and improvement of autonomous vehicles will be helped along. This depends, however, on there not being a group of people whose risks of injury are bound to be raised by the introduction of autonomous cars. If there was to be such a group, this could pose a major ethical problem for the introduction of autonomous cars.

## A Duty to Intervene

An alternative would be to hold the users of autonomous cars responsible for possible accidents. One version of doing so could be based on a duty of the user to pay attention to the road and traffic and to intervene when necessary to avoid accidents. The liability of the driver in the case of an accident would be based on his failure to pay attention and intervene. Autonomous vehicles would thereby lose much of their utility. It would not be possible to send the vehicle off to look for a parking place by itself or call for it when needed. One would not be able to send children to school with it, use it to get safely back home when drunk or take a nap while traveling. However, these matters are not of immediate ethical relevance.

As long as there is some evidence that a system in which people must intervene would do noticeably better in terms of number of accidents than one in which autonomous vehicles are left to themselves there is much to be said in favour of such a duty. If the introduction of autonomous vehicles reduces accidents by fifteen percent, and a duty to intervene for the “driver” would lower the death rate by another fifteen, that would seem to create a moral obligation on drivers to be on the lookout for possible failure. (Of course, this duty to interfere would still have to be limited to cases in which the driver could have been reasonably expected to anticipate the danger and react in time.) Also, it would also give the technology an opportunity to develop gradually. Autonomous driving could slowly evolve, going from the current level of automation through a number of intermediate stages to fully autonomous cars. On the downside, self-driving cars would, in such a scenario, not be useable by physically impaired, disabled or elderly people.

But once development has reached the stage of truly autonomous cars which drive at least as safely as the average human driver, we have to ask the question whether we can realistically expect the user to effectively intervene in emergencies. It is, of course, a question that can only be conclusively answered on the basis of empirical data.

However, it might be possible to take an educated guess. Accidents are usually not easily foreseeable—especially if there is no driver that might be noticeably tired, angry or distracted. Therefore, it will probably be difficult to recognize dangerous situations which the autonomous vehicle might be ill equipped to manage, and even harder to intervene in time. Of course, much will depend on what kind of cases we are talking about. If the problem in which the driver must intervene tend to be foreseeable (if there is, for example, some sort of timely warning sign given by the vehicle), this is not a problem. But once we are talking about fully autonomous cars which drive as safely as the average person, such a predictability of dangerous situations seems unlikely and unrealistic. Moreover, accidents could not only happen because persons fail to override the system when they should have, but also because people override it when there really was no danger of the system causing an accident (Douma and Palodichuk 2012). As the level of sophistication of autonomous cars improves, the possibility of interventions by the driver might cause more accidents than it helps to avoid.

But even assuming such intervention was possible, if the person in question were sufficiently focussed, one might still question if people would be able to keep up the necessary attention over longer periods of time. Fully autonomous vehicles will only be market-ready (we assumed) once they drive more safely than the average human driver does. According to a German statistic, which refers to accidents with damage to persons between 2005 and 2009, cars had on average about one accident in 1.46 Million Kilometres. (Vorndran 2010) If the number of accidents with autonomous vehicles is even lower, it seems implausible that the otherwise idle user will be able to stay completely focused and searching for a possible risk of an accident which might occur on average once every 2 million kilometres or so. One may speculate that few people will have the necessary ability to concentrate under these circumstances, and empirical findings corroborate this.^7^ Of course, a driver may be aware of and responsible for his level of alertness. Drivers might be required to pull over if they are not alert, driver alertness monitoring technology might help with that. To us, the viability of such an approach seems questionable; but in the end, we will have to wait for empirical data. As long as a duty to monitor the road and intervene in dangerous situations proves to decrease accidents compared to purely autonomous driving, such an approach is legitimate.

However, if it becomes clear that humans are typically not able to effectively intervene when necessary to avert imminent accidents involving sophisticated autonomous vehicles, it becomes problematic to blame a person for an accident just because he did not – indeed could not prevent it. As a rule, such allocation of responsibility is connected to notions of responsibility and blameworthiness. The person in question is held responsible by the state to have, through his inattention, allowed an accident to happen; causing death or injury of another human being. This is legitimate as long as one can actually expect the person in question to foresee the danger and prevent it. However, if human beings typically lack the necessary speed of reaction and attention span to do such a thing when using autonomous vehicles, this causes certain difficulties. As von Hirsch and Simester argue in the context of the moral limitations of the criminal law, official moral condemnations of an activity and an actor generate certain truth-constraints:

“[An] official moral condemnation of activity and actor generates a truth-constraint. When labelling conduct as wrongful, and when labelling those it convicts as culpable wrongdoers, the state should get it right. There are reasons to do this even apart from the intrinsic wrongness of telling untruths. For one thing, people have a moral entitlement not to be designated, officially, as miscreants when they do no wrong. Morally speaking, those whose conduct is not reprehensible ought not to be convicted and made punishable. […] A conviction for φing [doing X] has the effect of designating D as a criminal (in respect of that particular offence), a designation communicated to the public as well as to D. Where φing [doing X] is not wrongful, this amounts to moral defamation by the state. The state is no longer telling the public the truth about D. People have, we think, a moral right not to be censured falsely as criminals, a right that is violated when one is convicted and punished as a criminal without having perpetrated culpable wrongdoing.” (Simester and von Hirsch 2011).

This argument holds true even if we look at it purely in terms of what is morally right. It is a form of defamation (which is not just a legal, but also a moral concept) if a person is publicly and wrongly blamed for causing the death of another human being. This is quite obvious when it comes to highly wrongful acts like murder, but it is also true with regard to acts that cause harm through negligence. It is wrong to publicly accuse someone as having caused the death of another person with his inattention if he never had a real chance to do what was supposedly his “duty”. Furthermore, unfounded defamations of this sort are particularly problematic when communicated with the authoritative voice of the state.

## Responsibility of the Driver as a Form of a “Strict Liability”^8^

One alternative would be an approach in which the person in charge of the autonomous vehicle has no duty (and possibly no way) of interfering, but still be considered morally responsible for possible accidents. The rationale behind this would be that he took the risk of using the vehicle, knowing and accepting that it might cause accidents. Using a car poses a risk—for the person himself and for others. The more we use cars (especially where it is not necessary), the more we put others at risk—even if we do our best to drive safely. It is a moral issue much overlooked by philosophers, a fact that Husak wonders about in his 2004 article on the subject. The switch from human-driven cars to autonomous ones is not likely to change that. Even if the switch reduces the likelihood of accidents, the number of people injured and killed by cars each year will probably stay significant. It does therefore not appear implausible to consider the user at least partly responsible for accidents which may be caused by his vehicle.

This responsibility can take at least two different forms. In one (*Scenario A*), the person in question is only held responsible for taking the risk of using a car. It is a risk taken daily by millions of people all over the country. And at least in the case of autonomous vehicles, the risk taken by every single one of them will be highly comparable. If the user were only to be responsible for taking the risk of using the vehicle, he would therefore share this responsibility with every other person in the country who does the same. From this perspective, they did not do something wrong in the sense of it being blameworthy but they did participate in a practice which carries risks and costs for others and it therefore is their responsibility to shoulder that burden.

This would speak in favour of a system in which the cost of any accident caused by a (well-maintained, up to date, non-tampered-with etc.) autonomous vehicle is shared by all the owners/users of such vehicles. A scheme like that might work like a tax or a mandatory insurance, possibly partly based on the number of miles driven per year.

Alternatively, we might focus the blame for accidents on the person using the vehicle at the time (*Scenario B*). He took the risk of using the car and he would be held personally responsible for any accidents that are caused by it. Of course, the person whose autonomous vehicle crashes did not do anything different from any other user of autonomous cars; he was simply unlucky. He also did better than people using “normal” cars – at least if we assume autonomous cars to be the safer alternative.

However, such an approach would still in a way reflect our current practise of ascribing responsibility for accidents. Drivers who cause an accident by not paying attention for a moment or driving a bit too fast certainly made a mistake. But it is a mistake most of us have made at some point. Most if not all of us had moments in which we failed to pay proper attention to the road while driving, or moments in which we found ourselves driving too fast. The fact that in some cases such behaviour has horrible effects turns something which we treat as a small mistake into something which we treat as a major moral failing. The logic behind this could, as we will see, be also applied to the case of the user of an autonomous vehicle.

To hold the driver responsible not only for making his “small mistake”, but also for the accident itself, we have to assume his bad luck to be morally relevant. It is a common assumption held among others by Thomas Nagel (1982). The arguments in favour of genuine moral luck have the following simple structure: First, an example is given in which the moral assessment is uncontroversial but partly dependent on chance. (someone drives too fast and runs into a child). Since the concrete results and consequences of what is done are beyond the control of the actor, it seems that the moral assessment of what he does is dependent on something beyond his control too. It is a matter of chance.

One such standard example is the following situation: A person drives slowly and responsibly, keeping to the traffic rules, he is not drunk, he is fully concentrated, but nevertheless a horrible accident takes place because a child he could not have seen in advance runs out into the street from behind a group of parked cars. It is impossible for him to stop before his car hits the child. The child dies. Nagel interprets this situation as follows. *“The driver, if he is entirely without fault, will feel terrible about his role in the event, but will not have to reproach himself. Therefore this example of agent*-*regret is not yet a case of moral bad luck.”* (Nagel 1982) Most ethicists agree on that point.

According to Nagel, chance only becomes morally relevant if the driver has done something wrong to begin with. So let us modify the situation by introducing only one additional element: he drove carelessly, for example, too quickly given the situation. The child runs into the street, he cannot stop the car. The child dies. He feels as miserable as he would have felt miserable in the first case, but now he is aware of the fact that if only he had driven carefully instead of carelessly, the child would probably still be alive. To make this point a bit more precise: let us assume that if he had driven 10 mph more slowly he could have stopped before the car hit the child, and given the situation there, careful driving in general would require one to drive 10 mph less. Nagel’s position is that in such a case it makes a moral difference whether he hit the child or not. Careless driving gives reason to blame oneself slightly, but if careless driving results in the death of a child, it gives reason to blame oneself greatly. Since the driver has no control over whether his careless driving results in an accident or not, this seems to be an example of genuine moral luck, i.e., an example for the assumption that luck or chance is morally relevant.

This position is based on the assumption that it is possible to draw a clear line between the blameless driver and the (at least partly) guilty one. However, to uphold the rules, to drive carefully and considerately only limits the risk for others. The risk of others being harmed will still not be reduced to null. Furthermore, as Husak (2004) points out, our callous attitude towards the use of cars (especially when it is not necessary), which costs tens of thousands of lives each year, might also be morally questionable. So we might still consider his behaviour blameworthy, at least to a small degree. For this very same reason we might blame a person using a sophisticated autonomous vehicle if it causes an accident-at least partly. He did decide to use a car, fully aware that he might hit another person, a child.

This means no driver could ever be “absolutely without fault” if his vehicle runs into another human being. It was a risk he knew about, a risk he took. Some sort of liability can always be morally justified when using dangerous vehicles like cars that have a chance of injuring others. Usually this might not be a major problem, but it is one for the Nagelian notions of moral luck, since according to him, bad luck is only morally irrelevant if the driver is “absolutely without fault”—which he never is.

We might conclude that any use of cars (both autonomous and human-driven) should be subject to moral luck. However, such a position seems ludicrous and even most proponents of moral luck (including Nagel) would not except it. The underlying problem lies at the heart of the concept of moral luck: there is not just good and bad, right or wrong. Some actions seem to be perfectly fine under a moral perspective. Other actions seem to some to be slightly deviant, though others consider them acceptable. Some comply with juridical laws, but seem doubtful, at least from a moral perspective. Yet others are in conflict with legal rules and at the same time seem to be morally acceptable or even obligatory. There is a continuum between absolutely right and outrageously wrong. To assert that at one end of the spectrum chance has no moral relevance whatsoever, and that if one deviates even the slightest degree from that point, chance then gains a tremendous moral relevance, is in itself inadequate and leads to absurd conclusions.

All things considered, scenario (*B)* does not appear a plausible position. In the end, it is more an example of what is wrong with the concept of moral luck^9^ and our current practise of ascribing blame in case of accidents, than a credible alternative for the attribution of responsibility in case of accidents of autonomous cars.

## Conclusion

We discussed who should be held responsible for accidents of fully autonomous cars from a moral stand point. Both the *duty to intervene* and a *responsibility of the driver as a form of a “strict liability*” seem like viable options.

In the case of a duty to intervene this depends on there being an actual chance for the driver to effectively anticipate and prevent accidents. If the average driver never had a real chance of preventing an accident (either in the particular case at hand or in principle) he should not be held responsible for it. Therefore this option seems more attractive to us as an interim solution for the period in which autonomous cars are first introduced and developed. Once the development of autonomous cars has reached a point where people cannot effectively intervene any more, a contra factual duty to do so would be morally indefensible. Also, a duty to intervene would keep autonomous cars from being useable by the blind, elderly, etc.

In the case of a *responsibility of the driver as a form of a “strict liability*”, scenario (A) is the more viable one. It is justifiable to hold users of autonomous cars collectively responsible for any damage caused by such vehicles–even if they had no way of influencing the cars behaviour. However, this responsibility should not exceed a responsibility for the general risk taken by using the vehicle. A tax or a mandatory insurance seems the easiest and most practical means to achieve that.

Assuming the implementation of autonomous cars would save lives, this by itself constitutes a powerful moral reason to limit the possible responsibilities of manufacturers to a point where it does not render the development of such cars too risky for the companies involved. Of course, manufacturers should not be freed of their liability in cases like the Ford Pinto, in which the manufacturers put the car on the market fully knowing that it had major safety defects, but considered rectifying those flaws too expensive. Also, a certain amount of responsibility for accidents not is only morally desirable in itself but also an important incentive for the continuous development and improvement of such cars.

## Footnotes

1 A more thorough analysis of whether we can expect autonomous cars to crash can be found in Goodall (2014).

2 There will always be design decisions that have to be made, which will improve the safety of vehicles in certain situations, while increasing risks in others. The clearest example of this is the positioning of the gas tank. *“Any possible placement of the fuel tank ‘causes’ some accidents and averts others. Respectable designers have tried every gas*-*tank location at one time or another…. All have been rejected at other times as unsafe.”* (Olson 1993) Similar decisions will probably have to be made in the Systems which make a car autonomous.

3 As already stated in the beginning of the article, we will assume that it will be possible to design autonomous vehicles, which cause fewer and less severe accidents than cars steered by the average driver.

4 For a more thorough analysis of the deontological criteria for dealing with risks in a legitimate manner, see Nida-Rümelin et al. (2012).

5 The trolley-problem is a much-employed ethical thought-experiment which was first introduced by Philippa Foot: “it may […] be supposed that he [the man in her example] is the driver of a runaway tram which he can only steer from one narrow track on to another; five men are working on one track and one man on the other; anyone on the track he enters is bound to be killed.” How should the driver decide? (Foot 1967).

6 It should, however, be mentioned that ethical problems of this sort might still play an important role in other questions surrounding autonomous cars. Certain pre-programmed reactions of an autonomous car in case of accidents might, for example, use some groups as means to an end.

7
*[…]this would require that a driver pay constant attention to the roadway, similar to when using cruise control. Drivers in semi*-*autonomous vehicles with lane*-*keeping abilities on an empty test track exhibited significant increases in eccentric head turns and secondary tasks during automated driving, even in the presence of a researcher. Twenty*-*five percent of test subjects were observed reading while the vehicle was in autonomous mode. Similar results have been found in driving simulator studies. The effect of automation on a driver’s attention level remains an open question, but early research suggests that a driver cannot immediately take over control of the vehicle safely. Most drivers will require some type of warning time. *(Goodall 2014) The experiments which are referred to were originally described in Llaneras et al. (2013) and Jamson et al. (2013).

8 We will use this legal term to refer to a moral stance according to which the user does not have any duty of intervention in case of an impending accident, but has some moral responsibility for the accident anyway.

9 For a more detailed analysis of moral luck see Nida-Rümelin (2007).
